# Functional characterization and proteomic analysis of *lolA* in *Xanthomonas campestri*s pv. *campestris*

**DOI:** 10.1186/s12866-019-1387-9

**Published:** 2019-01-21

**Authors:** Chao-Tsai Liao, Ying-Chuan Chiang, Yi-Min Hsiao

**Affiliations:** Department of Medical Laboratory Science and Biotechnology, Central Taiwan University of Science and Technology, Taichung, Taiwan

**Keywords:** Lipoprotein, Proteomic analysis, Virulence

## Abstract

**Background:**

The gram-negative *Xanthomonas campestris* pv. *campestris* is the pathogenic bacterium that causes black rot disease in crucifers. The virulence determinants of this bacterium include extracellular enzymes, exopolysaccharides, and biofilm formation. Here, one transposon mutant of *X. campestris* pv. *campestris* strain 17 that affects biofilm formation was isolated, and subsequent analyses led to the identification of the *lolA* gene, which encodes an outer membrane lipoprotein chaperone.

**Results:**

The *lolA* mutant exhibited significant reductions in bacterial attachment, extracellular enzyme production, virulence, and tolerance in the presence of myriad membrane-perturbing agents. These phenotypic changes of the mutant could be complemented to the wild-type level through the intact *lolA* gene. Proteomic analysis revealed that 109 proteins were differentially expressed after *lolA* mutation. These differentially expressed proteins were categorized in various functional groups and were mainly associated with the membrane component, were involved in transport, and contained receptor activity. Through reverse transcription quantitative real-time polymerase chain reaction (RT-qPCR) analysis, deletion of *lolA* was determined to have caused significantly reduced expression of genes that encode the major extracellular enzymes, the biofilm-related proteins, and the virulence-related proteins. The RT-qPCR analysis also indicated that the expression of several genes that encode putative outer membrane lipoproteins and TonB-dependent receptors was reduced after *lolA* mutation.

**Conclusions:**

This is the first report to define the *lolA* gene as a virulence factor and to contribute to the functional understanding of, and provide new information concerning, the role of *lolA* in *Xanthomonas*. Furthermore, the results of this study provide and extend new insights into the function of *lolA* in bacteria.

**Electronic supplementary material:**

The online version of this article (10.1186/s12866-019-1387-9) contains supplementary material, which is available to authorized users.

## Background

*Xanthomonas* is a large genus comprised of 27 species of gram-negative bacteria that cause severe diseases in approximately 400 host plants that include crops of major economic value [1]. Within the *Xanthomonas* genus, *Xanthomonas campestris* is the most dominant species, having at least 141 pathovars that infect multifarious plants with agronomical importance [2]. *X. campestris* pathovar *campestris* infects cruciferous plants and causes black rot disease, which may be the most problematic disease to affect crucifers worldwide [2]. *X. campestris* pv. *campestris* is responsible for major yield and quality losses in *Brassica* crops—most of the hosts of this pathogen are members of *Brassica oleracea* such as cabbage, cauliflower, broccoli, and kale [2–4].

The virulence of plant pathogenic bacteria often depends on factors secreted and surface compounds that enable bacteria to infect and successfully grow in host tissue. *X. campestris* pv. *campestris* employs myriad virulence factors to invade its host, such as extracellular enzymes (cellulase, mannanase, pectinase, and protease) secreted by the type II secretion system, effector proteins exported by the type III secretin system, and exopolysaccharides [5–7]. The extracellular enzymes can degrade plant cell components and induce plant tissue maceration, and the effector proteins injected into the plant cells can interfere with cell physiology and plant immunity [3, 5, 7]. The exopolysaccharides can obstruct the xylem vessels, which causes tissue necrosis and leaf wilting [2]. Furthermore, exopolysaccharides have been determined to be involved in the formation of biofilm, a bacterial population in which bacteria attach to each other or to biotic or abiotic surfaces [5, 6]. Adhesion of plant pathogenic bacteria to surfaces is critical for invasion of the host, and the ability of bacteria to form and disperse biofilm may have implications for survival on leaf surfaces and within host plants [8]. In *X. campestris* pv. *campestris*, several factors reported to influence biofilm formation and bacterial adhesion also have roles in pathogenicity, namely extracellular mannanase ManA [6], GGDEF domain protein XC_0249 [9], guanylate cyclase XC_0250 [9], isocitrate dehydrogenase encoded by *icd2* [10], major outer membrane protein MopB [11], the RavA/RavR two-component system [12], tail-specific protease encoded by *prc* [13], and UTP-glucose-1-phosphate uridylyltransferase encoded by *galU* [14].

To discover novel biofilm-related genes in *X. campestris* pv. *campestris*, we previously employed Tn*5*-based random mutagenesis to construct a mutant library of *X. campestris* pv. *campestris* strain 17 (hereinafter, Xcc17), and the adhesion ability of the generated mutants was tested to identify genes whose loss of function caused alterations in bacterial attachment and biofilm formation [13]. In this study, one mutant designated as H27 showing reduced bacterial attachment was characterized. The mutant strain H27 was determined to have a transposon inserted in the locus_tag AAW18_RS09800, which was annotated to encode an outer membrane lipoprotein chaperone LolA in the recently completed genome sequence of Xcc17 (GenBank accession no. NZ_CP011946) [15].

The lipoprotein outer membrane localization (Lol) system plays a central role in the sorting of lipoproteins to the outer membrane [16]. In *Escherichia coli*, five proteins, namely LolABCDE, constitute the Lol system that is responsible for sorting and localizing lipoprotein, and all Lol proteins are considered essential for cell growth [16–20]. First, the ATP binding cassette (ABC) transporter LolCDE releases outer membrane-directed lipoproteins from the inner membrane. Subsequently, the released outer membrane-specific lipoproteins form a complex with the periplasmic chaperone LolA. Finally, the lipoproteins are transferred from LolA to the outer membrane receptor LolB and localized to the outer membrane [16–20]. The *lol* homologues can be found in many gram-negative bacteria, but the conservation of individual genes varies [16–18]. LolCDE homologues in most γ-proteobacteria consist of one copy each of membrane subunits LolC and LolE, and two copies of ATP binding subunit LolD [16]. While in α-, β-, and some γ-proteobacteria such as *Francisella tularensis*, *Legionella pneumophila*, and *Acinetobacter baumannii*, the ABC transporter was found to have a single membrane subunit that contains sequence features found in both LolC and LolE of *E. coli*, and named LolF [16, 21]. In *E. coli*, the ABC transporter is encoded by an operon with a *lolCDE* gene order, while *lolFD* organization was found in members of the α-, β-, and in some γ-proteobacteria, such as *F. tularensis*, *L. pneumophila*, and *A. baumannii* [16, 21]. The *lolB* gene is found only in β- and γ-proteobacteria [18]. Despite the fact that Lol homologues are conserved in a range of gram-negative bacteria, only the Lol proteins of *E. coli* have been studied in detail. To date, only the five Lol proteins of *Pseudomonas aeruginosa* have been reported to be involved in the lipoprotein sorting to the outer membrane, as in the case of *E. coli* lipoproteins [22]. Thus, the objective of this study was to investigate the physiological roles of *lolA* in Xcc17. This is the first time that *lolA* has been characterized in *Xanthomonas*.

## Methods

### Bacterial strains and culturing conditions

*E. coli* DH5α ECOS™ 101 (Yeastern Biotech, Taipei, Taiwan) was the host for DNA cloning. The *lolA* mutant H27 was derived from *X. campestris* pv. *campestris* strain Xcc17 (a virulent wild-type strain isolated in Taiwan [23]) through transposon mutagenesis. The bacteria strains were routinely cultured in Luria–Bertani medium [24] at 37 °C for *E. coli* and 28 °C for *X. campestris* pv. *campestris*. The XOLN medium contained basal salts, 0.625 g/L tryptone, and 0.625 g/L yeast extract [25]. Carbon sources used were glucose (2%) and glycerol (2%) as necessary. Liquid cultures were shaken at 180 rpm. Solid media contained 1.5% agar. Antibiotics were added at the following concentrations when necessary: ampicillin (50 μg/mL), kanamycin (50 μg/mL), and tetracycline (15 μg/mL).

### Recombinant DNA techniques

The Wizard® Genomic DNA Purification Kit (Promega) and the Gene-Spin™ Miniprep Purification Kit (Protech) were used to prepare bacterial genomic DNA and plasmid DNA, respectively. Polymerase chain reaction (PCR) was performed as previously described [26]. Standard protocols as described previously [27] were used for agarose gel electrophoresis, DNA ligation, restriction digestion, and *E. coli* transformation. Transformation of *X. campestris* pv. *campestris* was achieved through electroporation [28]. The DNA sequences were determined by Mission Biotech Co., Ltd. (Taipei, Taiwan).

### Transposon mutagenesis and insertion site identification

The EZ-Tn*5*™ < R6Kγ*ori*/KAN-2 > Tnp Transposome Kit (Epicentre) was used to produce the *X. campestris* pv. *campestris* transposon mutants in accordance with the previously described method [13]. One transposon mutant, designated as H27, exhibiting reduced attachment was selected for further characterization. The site inserted by transposon in H27 was identified using the rescue cloning method using the TransforMax™ EC100D™ *pir*^+^ electrocompetent *E. coli* (Epicentre), as previously described [29].

### Complementation of the *lolA* mutant

For complementation of the *lolA* mutant, a 690-bp *Bam*HI/*Eco*RI fragment encompassing the upstream 60-bp fragment plus the entire coding region of *lolA* was amplified by PCR using primers lolA-F (5′-GGATCCGCGCTCGCCCATTCATCTAT-3′, *Bam*HI restriction site underlined) and lolA-R (5′-GAATTCCTACTGCGCGTCGCCCACCA-3′, *Eco*RI restriction site underlined) and ligated into cloning plasmid yT&A (Yeastern), yielding pTlolA. After sequence verification, the 690-bp *Bam*HI/*Eco*RI fragment of pTlolA was excised and cloned into the *Bam*HI-*Eco*RI sites of the broad-host-range plasmid pRK415 [30]. The construct pRKlolA was obtained and transferred into the *lolA* mutant strain H27 through electroporation, resulting in the complemented strain H27(pRKlolA). In parallel, the empty vector pRK415 was transferred into Xcc17 and H27, yielding Xcc17(pRK415) and H27(pRK415) for comparison.

### Attachment assay, pathogenicity test, and extracellular enzyme activity analysis

Bacterial attachment and pathogenicity test were done according to previously described procedures [13]. The experiments were performed at least three times. For attachment evaluation, the 96-well polystyrene microtiter plates (Nunc) were used. For virulence assay, the disease symptoms in cabbage (*B. oleracea* var. capitata) were photographed and lesion lengths were measured 10 days after inoculation.

Extracellular enzyme activity was determined according to previously described methods [31] with minor modifications. Briefly, 3 μL of overnight culture (OD_550_ = 1) was deposited onto the surface of agar plates containing the appropriate substrates: carboxymethyl cellulose (0.5%, substrate for cellulase), locus bean gum (0.2%, for mannanase), sodium polypectate (0.2%, for pectinase), and skim milk (1%, for protease). After 2 days of incubation, enzyme activity was determined as described previously [31]. Each experiment was performed at least in triplicate.

### Stress tolerance assay

For the stress tolerance assay, bacteria were grown overnight and inoculated into fresh XOLN medium containing glycerol to obtain an initial OD_550_ of 0.35 in the presence or absence of one type of stress condition, namely EDTA (0, 0.2, and 0.5 mM), polymyxin B (0, 2, and 20 μg/mL), and SDS (0, 0.0075, and 0.01%). After incubation at 28 °C with shaking (180 rpm) for 24 h, OD_550_ values were measured to determine the growth of each strain. Each treatment was performed at least three times.

### RNA extraction, reverse transcription (RT), and quantitative real-time PCR (qPCR)

Total RNA was extracted from cultures of *X. campestris* pv. *campestris* strains grown to the mid-exponential phase (OD_550_ = 0.6) in XOLN medium plus 2% glycerol by using the RNeasy Mini Kit (Qiagen). To eliminate genomic DNA contamination, the isolated RNA was treated with DNase by using the RNase-Free DNase Set (Qiagen) to remove residual DNA. The RT reaction was performed using the iScript™ cDNA Synthesis Kit (BIO-RAD) according to the manufacturer’s instructions. Briefly, the first-strand cDNA was synthesized from 1 μg of RNA by using random hexamer primers and reverse transcriptase supplied with the iScript™ cDNA Synthesis Kit. The reaction conditions included primer annealing at 25 °C for 5 min, RT at 46 °C for 20 min, and subsequently reverse transcriptase inactivation at 95 °C for 1 min. Subsequent qPCR was performed using iQ™ SYBR® Green Supermix in a CFX96 Real Time PCR system (BIO-RAD). The sequences of primer sets of the tested target genes are listed in Additional file 1: Table S1. The *16S rRNA* gene was used as the reference gene. The PCR amplification conditions were as follows: 3 min at 95 °C, and 40 cycles of 10 s at 95 °C, 10 s at 60 °C, and 30 s at 72 °C. All qPCRs were performed in triplicate. The amount of transcripts was presented as the n-fold difference relative to the reference gene (2^*-*Δ*Ct*^ where Δ*Ct* refers to the difference in threshold cycles between the target and reference genes). The results were indicated in relation to wild-type 2^-Δ*Ct*^ levels, which were referred to as 1.

### Extraction of outer membrane proteins

The outer membrane fraction of *X. campestris* pv. *campestris* was extracted according to a procedure described previously [32] with some modifications. Briefly, the *X. campestris* pv. *campestris* strains were cultured in XOLN–glycerol medium. When the strains had grown to the mid-exponential phase (OD_550_ = 0.6), the cells were harvested through centrifugation (10,000 *g* at 4 °C for 5 min). Each membrane preparation was obtained from the same number of cells. The cell pellets were rinsed and suspended in 10 mM HEPES buffer (pH 7.4) and sonicated on ice by using 600-μm glass beads (two bursts, 3 min). Following sonication, the unbroken cells and debris were removed through centrifugation at 15,600 *g* for 2 min at 4 °C. The supernatant was subsequently transferred to a new centrifuge tube, and the membrane-containing fraction (having both the cytoplasmic membrane and outer membrane) was pelleted through centrifugation at 15,600 *g* for 30 min at 4 °C. The pelleted membrane-containing fraction was re-suspended in 10 mM HEPES (pH 7.4) and 2% lauroyl–sarcosine and incubated at room temperature for 30 min with intermittent mixing to solubilize the cytoplasmic membrane fraction. The sarcosine–insoluble outer membrane fraction was collected through centrifugation at 15,600 *g* for 2 h at 4 °C. The resulting pellets were washed once using 10 mM HEPES without disturbing the pellets and subsequently re-suspended in the same buffer. The protein concentration of sarcosine–insoluble outer membrane fraction in the final preparation was determined using the Bradford method.

### Gel electrophoresis and in-gel digestion of outer membrane proteins

Equal amounts of outer membrane proteins (20 μg) were loaded and resolved on 12.5% SDS-PAGE, and then the VisPRO 5 min Protein Stain Kit (Visual Protein, Taiwan) was used for gel staining. In-gel digestion was performed according the methods described previously [33]. Briefly, each lane was cut into five slices, and the gel piece was washed/dehydrated three times in 50 mM ammonium bicarbonate (pH 7.9)/50 mM ammonium bicarbonate (pH 7.9) and 50% acetonitrile. Subsequently, cysteine bonds were reduced using 10 mM dithiothreitol for 1 h at 56 °C and alkylated using 25 mM iodoacetamide in the dark for 45 min at room temperature. After two subsequent wash/dehydration cycles, the gel pieces were dried for 10 min in a vacuum centrifuge and rehydrated in 50 mM ammonium bicarbonate (pH 7.9) containing 6.25 ng/μL sequencing-grade trypsin overnight at 25 °C. After trypsin digestion, peptides were extracted once in 100 μL of 1% formic acid and subsequently extracted twice in 100 μL of 50% acetonitrile in 5% formic acid. The volume was reduced to 50 μL in a vacuum centrifuge prior to mass spectrometry analysis.

### Mass spectrometry analysis

Tryptic peptides generated as described in the preceding section were analyzed through nano liquid chromatography–tandem mass spectrometry (nLC-MS/MS) using an Ultimate 3000 nLC system (Dionex) connected to an LTQ Orbitrap mass spectrometer (Thermo Fisher Scientific). The nLC-MS/MS solvents were 0.05% formic acid in double distilled water (solvent A) and 80% acetonitrile in aqueous 0.05% formic acid (solvent B). For nLC separation, the tryptic peptides were first injected and trapped on a precolumn (Pepmap C18 cartridge, 5 mm × 300 μm i.d., Dionex) at a flow rate of 30 μL/min in 98% solvent A and 2% solvent B. The samples were subsequently transferred onto an analytical C18 column (20 cm × 75 μm i.d. fused silica column custom packed with 3 μm 120 Å ReproSil Pur C18 aqua, Dr. Maisch-GmbH) and separated at a flow rate of 300 nL/min for 60 min in a 10–40% solvent B gradient. The separated peptides were ionized at 1.7 kV using a Nanomate Triversa Chip–based nanospray source using a Triversa LC coupler (Advion). Intact peptide mass spectra and fragmentation spectra were acquired using an LTQ Orbitrap XL mass spectrometer (Thermo Fisher Scientific). Intact masses were measured at a resolution of 50,000 in the ion cyclotron resonance cell by using a target value of 1 × 10^6^ charges. In parallel, following a Fourier transform prescan, the top 5 peptide signals (charge states 2+ and higher) were subjected to MS/MS in the linear ion trap (3 amu isolation width, 30 ms activation, 35% normalized activation energy, Q value of 0.25, and a threshold of 5000 counts). Dynamic exclusion was applied using a repeat count of 1 and an exclusion time of 30 s.

### Database searching, protein identification and quantification, and bioinformatics analysis

BioWorks 3.3 (Thermo Fisher Scientific), a suite of layered software applications for protein identification and quantification, was used to interpret the acquired MS/MS spectra. The MS/MS spectra were searched against the *X. campestris* pv. *campestris* strain 8004 database from UniProt (4252 sequences; 1,445,974 residues) by using SEQUEST (version 27, rev. 12), which is part of the BioWorks 3.3 data analysis package. The MS/MS spectra were searched with a maximum allowed deviation of 10 ppm for the precursor mass and 0.6 Da for fragment masses. Methionine oxidation was allowed for variable modifications, cysteine carbamidomethylation was allowed for fixed modifications, two missed cleavages were allowed, and the minimum number of tryptic termini was 1. After searching the database, the DTA and OUT files were imported into Scaffold (versions 1.07 and 2.01) (Proteome software). Scaffold was used to organize the data and validate peptide identification by using the Peptide–Prophet algorithm, and only identifications with a probability of > 95% were retained. Subsequently, the Protein–Prophet algorithm was applied and protein identifications with a probability of > 99% with 1 or 2 peptides in at least one of the samples were retained. Proteins that contained similar peptides and could not be differentiated based on MS/MS analysis alone were grouped together. Protein quantification was estimated using spectral counting [34, 35]. Briefly, the spectral counts (the number of MS/MS associated with an identified protein) of each identified proteins were extracted from the Peptide–Prophet files and exported to Microsoft Excel software for downstream calculation. First, the spectral counts of identified protein were normalized by total spectrum for each sample. Then, the spectral count ratio (*lolA* mutant/wild type) was calculated. Finally, the obtained spectral count ratio was used for comparison values for identifying differentially expressed proteins between the wild type and *lolA* mutant. In this study, proteins with a spectral count ratio > 2 or < 0.5 (two-fold change between wild type and mutant) were designated as differentially expressed proteins.

Sequences of differentially expressed proteins were retrieved using their accession numbers in FASTA format from the UniProt database (http://www.uniprot.org/) and submitted to several web-based prediction tools to predict protein subcellular localization and secretion. The TMHMM Server v. 2.0 (http://www.cbs.dtu.dk/services/TMHMM/) was used to predict the transmembrane helices in proteins. The SignalP 4.0 Server (http://www.cbs.dtu.dk/services/SignalP/) was used to predict signal peptides [36]. Non-classical protein secretion was analyzed using the SecretomeP 2.0 Server (http://www.cbs.dtu.dk/services/SecretomeP/) [37]. Bacterial specific twin-arginine signal-peptide containing proteins were predicted using the TatP 1.0 Server (http://www.cbs.dtu.dk/services/TatP/) [38]. Gene Ontology (GO) term annotation and enrichment analysis were carried out using DAVID Bioinformatics Resources 6.8 (https://david.ncifcrf.gov/tools.jsp) [39]. The top ten categories were selected and present in Figures.

### Statistical analysis

Values are the means of three replications per experiment. Each experiment was performed at least three times. Student’s *t* test was used to determine the statistical significance of differences between means. *P <* 0.05 was considered statistically significant.

## Results

### Disruption of *lolA* reduces bacterial attachment

**Fig. 1:**
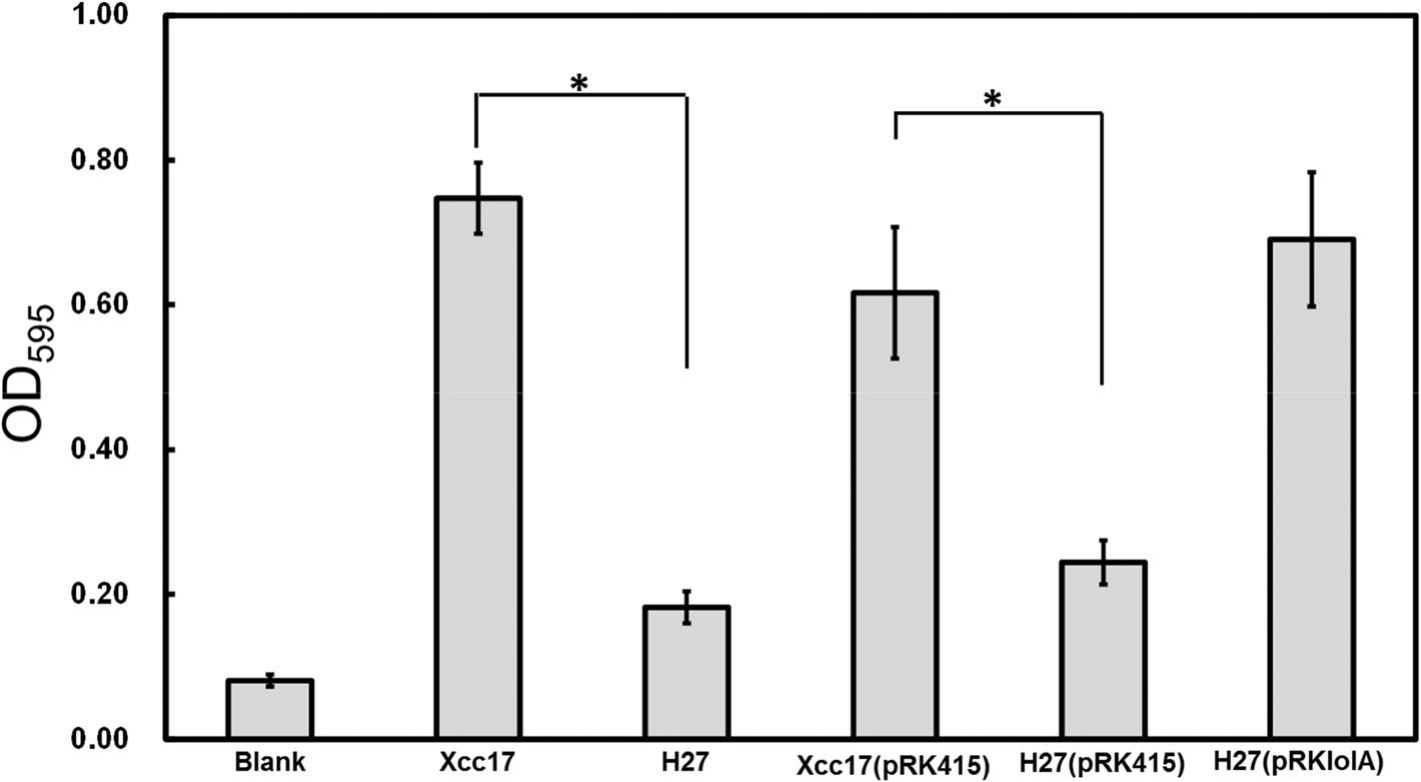
Effects of mutation of *lolA* on cell attachment to polystyrene surfaces in *X. campestris* pv. *campestris*. Strains to be assayed were grown overnight, cells were washed and diluted using fresh XOLN medium supplemented with glucose, and were tested as described in the Methods section. Xcc17: wild type; H27: *lolA* mutant; Xcc17(pRK415) and H27(pRK415): Xcc17 and H27 carrying empty vector pRK415; H27(pRKlolA): complemented strain. The XOLN medium supplemented with glucose was used as a blank. Values presented are the mean ± standard deviation (*n* = 3). Significance was determined using the Student *t* test. The asterisk (*) indicates *p* < 0.05

Previously, we subjected the *X. campestris* pv. *campestris* wild-type strain Xcc17 to transposon mutagenesis and subsequently screened approximately 1000 transposon mutants for bacterial attachment ability and biofilm formation [13, 29]. One mutant, H27, which exhibited reduced attachment ability, was characterized in this study. The mutant strain H27 exhibited reduced bacterial attachment compared with the parental strain Xcc17 as depicted in Fig. 1. No significant differences in growth rates or final yields were observed between Xcc17 and H27 when cells were cultured in the basal XOLN medium containing glucose or glycerol (data not shown), which indicated that the ability to use these carbon sources was unaffected.

Rescue cloning (Epicentre) was used to determine the transposon insertion site in H27. The transposon was discovered to have been inserted between positions 2,239,207 and 2,239,208 in the genome sequence of Xcc17 [15]. The transposon-inserted gene (locus_tag AAW18_RS09800, encoding an outer membrane lipoprotein chaperone LolA) was 630 bp in length and located at position 2,238,711–2,239,340 in the Xcc17 genome sequence [15]. The genes encompassing *lolA* were AAW18_RS09795 (encoding a DUF3857 domain-containing protein) and AAW18_RS09805 (encoding a hypothetical protein). AAW18_RS09795 was located upstream of *lolA* in the same direction with a 106-bp intergenic space, and AAW18_RS09805 was located downstream of *lolA* in the opposite orientation with a separation of 118 bp. The orientation and intergenic regions of *lolA* and its flanking genes indicated that transposon insertion in *lolA* does not cause a polar effect.

To further confirm that the altered attachment behavior exhibited by H27 was because of the insertion of transposon in *lolA* gene, a complemented strain named H27(pRKlolA) was generated by introducing the *lolA*-expression plasmid pRKlolA into the mutant strain H27. Bacterial attachment was compared, and Xcc17 and H27 carrying pRK415 (empty vector) were used for controls. The results indicated that pRKlolA (with the cloned *lolA* gene) could restore attachment ability to the wild-type level, but the empty vector pRK415 could not (Fig. 1**)**. These findings indicated that the transposon insertion in H27 was most likely not a polar mutation; moreover, the insertion inactivation of the *lolA* gene affected the bacterial attachment exhibited in this mutant rather than the malfunction of other genes flanking *lolA*.

### The gene *lolA* is involved in virulence

**Fig. 2:**
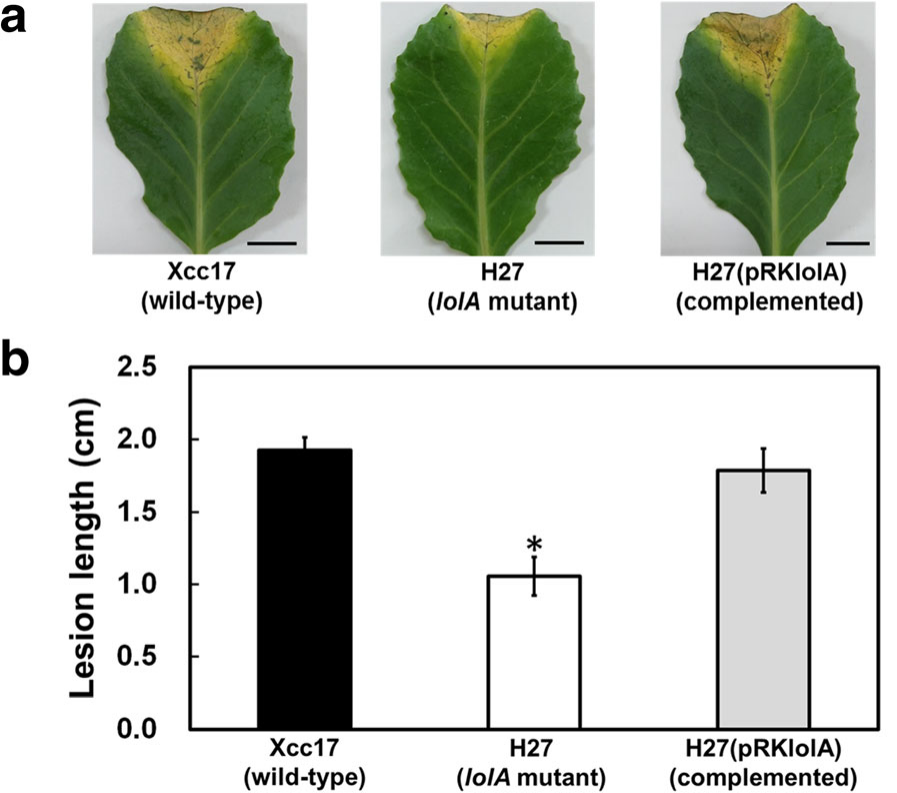
Effects of mutation of *lolA* on virulence of *X. campestris* pv. *campestris* in cabbage. **a** Black rot symptoms caused by *X. campestris* pv. *campestris* strains on inoculated leaves of host cabbage plant. Photographs were taken on day 10 after inoculation. **b** Average lesion lengths caused by *X. campestris* pv. *campestris*. Values presented are the means ± standard deviations from three repeats, each with six leaves. Significance was determined using the Student *t* test (* indicates significance at *p* < 0.05)

As several factors having a role in bacterial attachment have been reported to involved in pathogenicity in *X. campestris* pv. *campestris* [10, 11, 13, 14], we aimed to determine whether the *lolA* mutation affected the virulence of *X. campestris* pv. *campestris*. To evaluate the role of *lolA* in pathogenicity, the virulence of the mutant was tested on host-plant cabbage by using the leaf-clipping method [13]. Ten days after inoculation, typical “V”-shape black rot symptoms were visible on leaves inoculated with the wild-type strain and the complemented strain (Fig. 2a). The lesion lengths were approximately 1.92 and 1.78 cm for Xcc17 (wild type) and H27(pRKlolA) (complemented) at 10 days after inoculation, respectively (Fig. 2b). Although mutant H27 induced disease symptoms (Fig. 2a), its consequent mean lesion length (1.06 cm) was significantly shorter than that caused by the wild type (Fig. 2b). These results demonstrated that *lolA* is required for the full virulence of *X. campestris* pv. *campestris* to affect the host plant.

### Mutation of *lolA* causes reductions in extracellular enzyme production

**Fig. 3:**
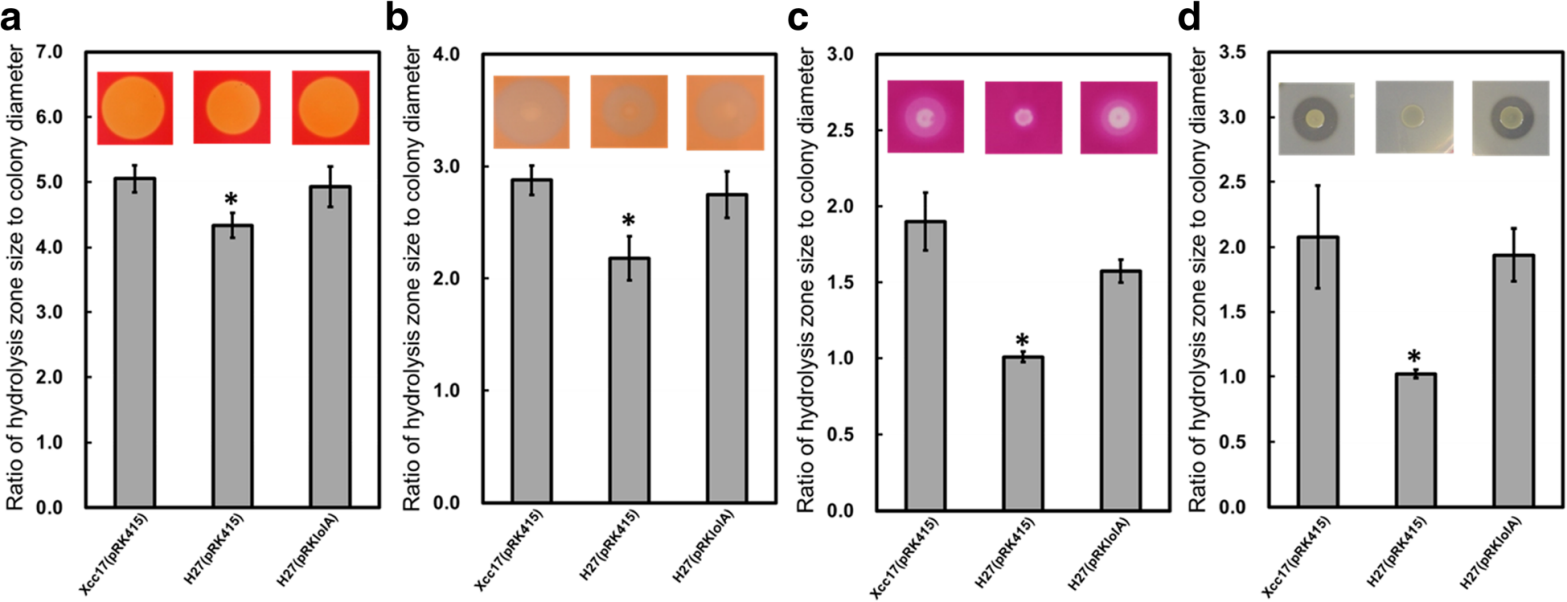
Effects of mutation of *lolA* on extracellular cellulase (**a**), mannanase (**b**), pectinase (**c**), and protease (**d**) activities in *X. campestris* pv. *campestris*. The extracellular enzyme activity was evaluated using the substrate-supplemented plate assay as described in the Methods section. Xcc17(pRK415): wild type; H27(pRK415): *lolA* mutant; H27(pRKlolA): complemented strain. The diameter of the colony and hydrolysis zone of each strain was measured after two days of incubation at 28 °C. The ratio of hydrolysis zone size to colony diameter is depicted. Values presented are the mean ± standard deviation (*n* = 3). Significance was determined using the Student *t* test (* indicates significance at *p* < 0.05)

The attenuated virulence observed in the *lolA* mutant (Fig. 2) suggested that this gene plays a role in the synthesis of multifarious pathogenic factors such as extracellular enzymes and exopolysaccharides. To assess the effect of *lolA* mutation on extracellular enzyme production, the hydrolytic activities of the *lolA* mutant were analyzed on a substrate–supplementary plate. The diameters of the colonies formed by different cells on the same plate were similar, whereas the diameters of the clearing zones, including the colonies formed by the *lolA* mutant, were significantly smaller than those observed for the wild type and complementary strain (Fig. 3). When exopolysaccharide production was tested, the exopolysaccharide yield of the *lolA* mutant exhibited no significant difference from that of the wild type (data not shown). These results indicated that the ability to synthesize extracellular enzymes (including cellulase, mannanase, pectinase, and protease) was impaired by mutation of the *lolA* gene, but mutation of *lolA* had no effect on exopolysaccharide production under our test conditions.

### Mutation of *lolA* influences the expression of genes involved in bacterial attachment and extracellular enzymes

**Fig. 4:**
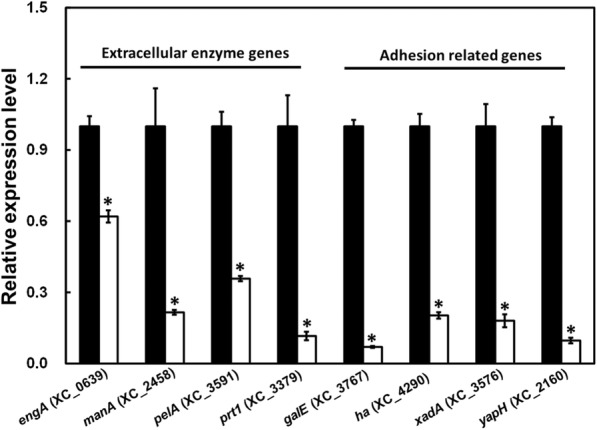
Effects of mutation of *lolA* on the expression of genes encoding extracellular enzymes or products associated with attachment. The expression level of extracellular enzyme genes (*engA*, *manA*, *pelA*, and *prt1*) and attachment-related genes (*galE*, *ha*, *xanA*, and *yapH*) in the wild-type strain Xcc17 (black bar) and *lolA* mutant strain H27 (white bar) was evaluated through RT-qPCR. The gene ID is based on *X. campestris* pv. *campestris* strain 8004 and listed in parentheses. The relative expression level of each gene in Xcc17 and H27 was normalized to its 16S rRNA content. Values presented are the mean ± standard deviation (*n* = 3). Significance was determined using the Student *t* test (* indicates significance at *p* < 0.05)

Because mutation of *lolA* reduced bacterial attachment (Fig. 1) and impaired virulence and extracellular enzyme production (Fig. 2 and Fig. 3), tests were conducted to determine if *lolA* contributes to the expression of genes related to these phenotypes and associated with pathogenicity. To test whether mutation of *lolA* affected the expression of genes related to bacterial attachment and extracellular enzyme synthesis, RT-qPCR was used to evaluate the transcription of genes that were selected because of their altered phenotypes mentioned earlier. Four genes that code for extracellular enzymes and have a role in virulence were selected: *engA*, which encodes major cellulase [7, 26], *manA*, which encodes major mannanase [6, 40], *pelA* which encodes major pectinase [7, 41], and *prt1*, which encodes major protease [7, 42]. In addition, the *galE* gene (encoding an UDP-galactose 4-epimerase), which was reported to have a role in bacterial attachment [43], and three putative adhesion-related genes that are present in the *X. campestris* pv. *campestris* genome (the *ha* gene, which encodes hemagglutinin, *xadA*, which encodes outer membrane adhesion, and *yapH*, which encodes autotransporter-like protein H) were also selected. RT-qPCR assay was performed to evaluate the transcription of these genes in the *lolA* mutant and wild-type strain. The results indicated that all of the tested genes were significantly downregulated at the transcription level in the *lolA* mutant H27 compared with the wild-type strain Xcc17 (Fig. 4). These results suggested that *lolA* mutation affects the expression of several virulence-related genes such as extracellular enzyme genes and adhesion-related genes.

### The gene *lolA* is required for tolerance to various stresses

**Fig. 5:**
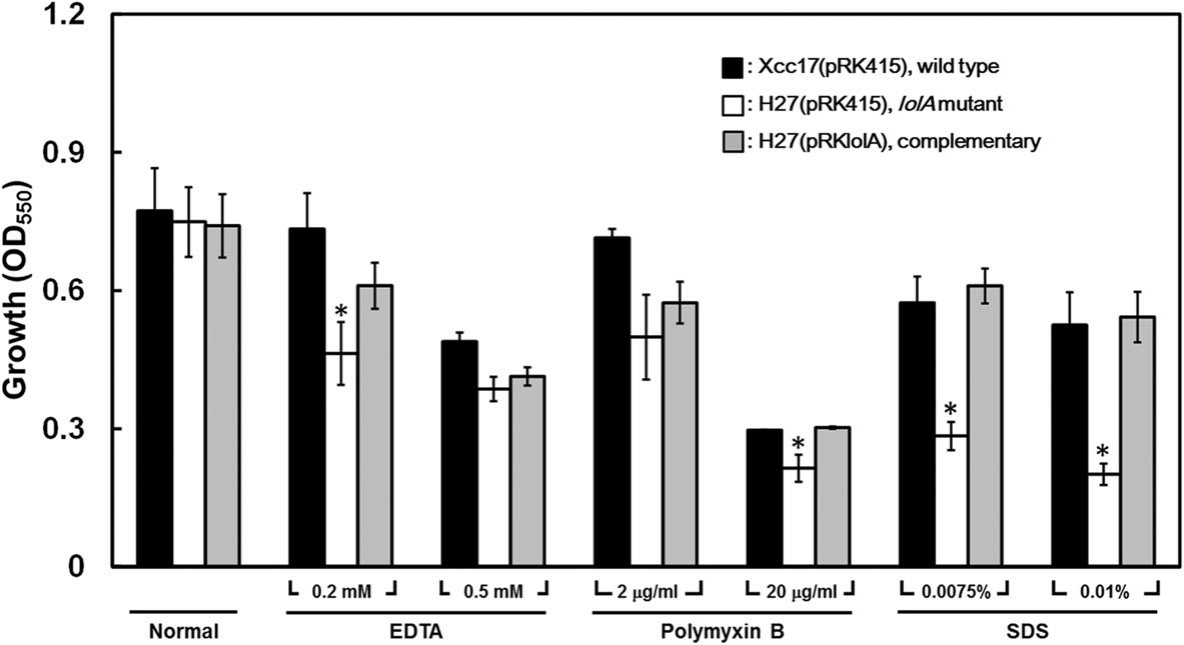
Effects of mutation of *lolA* on stress tolerance. Cells were grown in XOLN medium with or without various stress conditions. Cell density was measured at OD_550_ after 24 h. Values presented are the means ± standard deviations from three repeats. The asterisk (*) indicates *p* < 0.05

In view of the general role of biofilm formation in protecting bacteria from harsh environment and promoting bacterial survival against stresses, the reduced bacterial attachment ability of the *lolA* mutant (Fig. 1) suggested that the *lolA* gene might have a role in stress tolerance. To explore whether *lolA* affects the ability of *X. campestris* pv. *campestris* to tolerate stress, the wild-type strain Xcc17(pRK415), *lolA* mutant H27(pRK415), and complemented strain H27(pRKlolA) were exposed to various treatments, namely EDTA, polymyxin B, and SDS, and growth ability was evaluated. As Fig. 5 depicts, the *lolA* mutant dramatically reduced *X. campestris* pv. *campestris* tolerance to all the tested stresses. No significant difference was observed in stress tolerance between the wild-type strain and the complemented strain. These results indicated that *lolA* is involved in stress tolerance in *X. campestris* pv. *campestris*.

### Mutation of *lolA* has broad effects on the *X. campestris* pv. *campestris* outer membrane proteome

**Fig. 6:**
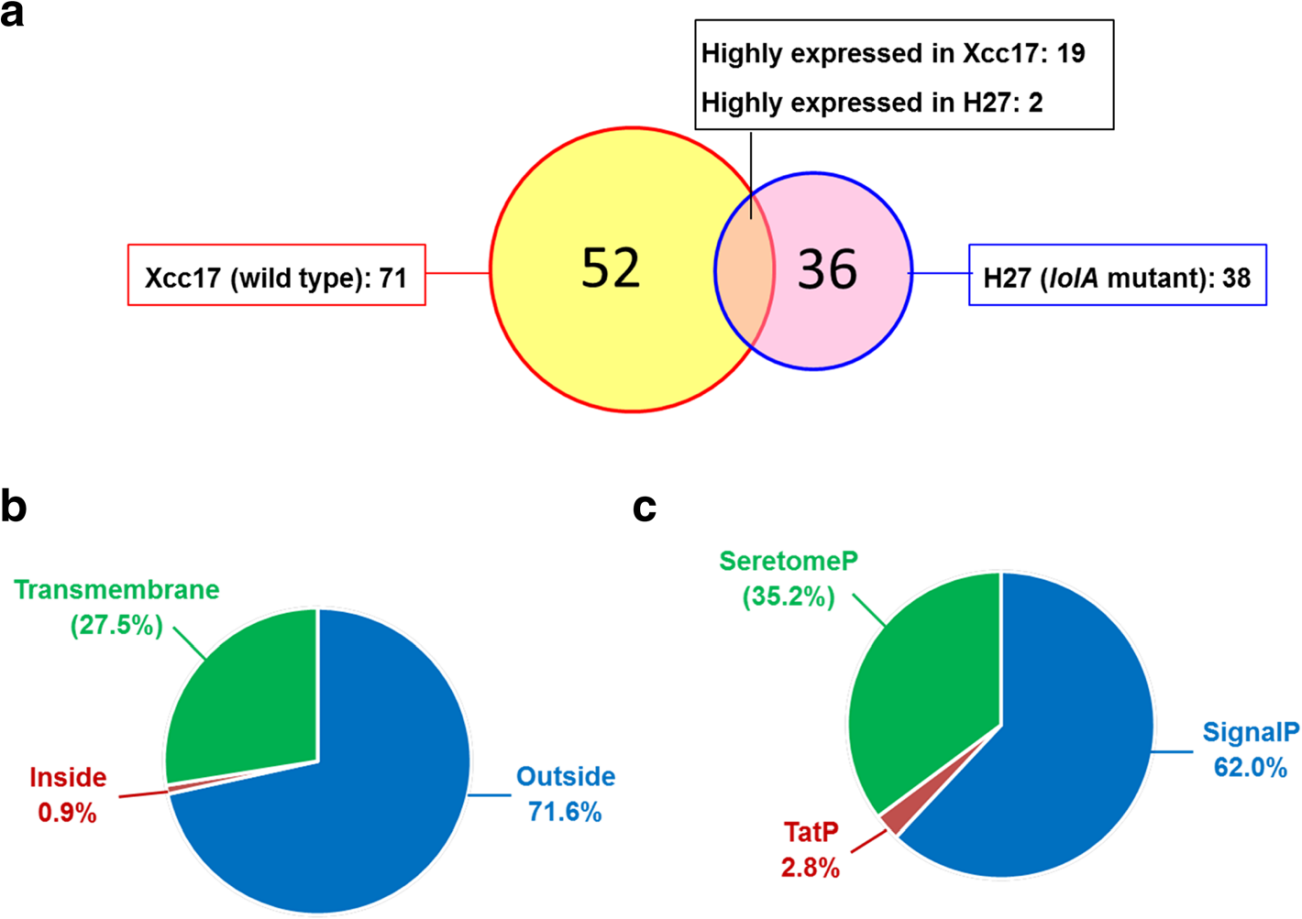
Differentially expressed proteins identified through proteomic analysis. Venn diagram (**a**) presents the numbers of differentially expressed proteins found in Xcc17 (wild type, red circle) and H27 (*lolA* mutant, blue circle). Pie chart (**b**) presents the protein location predicted using TMHMM. Pie chart (**c**) presents the signal peptide predicted by SignalP, SecretomeP, and TatP. Detailed information concerning differentially expressed proteins that were identified can be found in Additional file 2: Table S3

The *X. campestris* pv. *campestris* LolA deduced from the gene (630 bp) contained 209 amino acids. It had an *N*-terminal signal sequence of 21 amino acids and a possible cleavage site, AFA^21^–G^22^A, as predicted by signal P software [36]. Removing the signal peptide would produce a mature protein with a theoretical molecular mass of 20,685 Da and a p*I* of 5.62 (calculated using the Compute pI/MW tool from ExPASy; http://www.expasy.org/). A search of the conserved domain revealed that it contained a LolA domain (PF03548) located at residues 32–197 (bit score: 197.92, *E*-value: 1.35e-63). The *X. campestris* pv. *campestris* LolA exhibited 27% identity and 49% similarity to LolA from *E. coli* (UniProtKB P61316, encoded by the gene *b0891* in *E. coli* strain K-12), and 31% identity and 51% similarity to LolA from *P. aeruginosa* (UniProtKB Q9I0M4, encoded by the gene *PA2614* in *P. aeruginosa* strain PAO1). The LolAs from *E. coli* and *P. aeruginosa* are involved in sorting and transporting lipoproteins destined for the outer membrane [22, 44]. Although the roles of *X. campestris* pv. *campestris* LolA in lipoprotein outer membrane localization were not experimentally demonstrated, we discerned that the *lolA* mutant was more sensitive to myriad membrane-perturbing compounds (Fig. 5). Hypothetically, we reasoned that *X. campestris* pv. *campestris lolA* inactivation might impair the localization of outer membrane lipoprotein and alter the outer membrane protein profile. To test this hypothesis and acquire further insights into additional physiological roles of *lolA* in *X. campestris* pv. *campestris*, we performed a quantitative proteome analysis of the outer-membrane-enriched fraction and compared the protein profiles of the wild-type Xcc17 and the *lolA* mutant H27. The proteomic analysis was carried out using one biological replicate for both wild type and *lolA* mutant. Through the preliminary comparison of the protein expression profiles of these two strains, a general picture of LolA function could be obtained. As listed in Additional file 2: Table S2, 204 and 188 proteins were identified in the wild type and *lolA* mutant, respectively. Among these identified proteins, 109 proteins were differentially expressed (fold change > 2.0); 71 of the 109 proteins were upregulated, and 38 were downregulated (Additional file 2: Table S3). Of these differentially expressed proteins, (i) 52 proteins were uniquely present in the wild type, (ii) 36 proteins were only present in the *lolA* mutant, and (iii) the presence of 19 and 2 proteins was higher in the wild type and *lolA* mutant, respectively (Fig. 6a and Additional file 2: Table S3). These differentially expressed proteins were further subject to bioinformatics analysis to predict their location and investigate their function.

To predict the secretion and location of these differentially expressed proteins, TMHMM, SignalP, SecretomeP, and TatP analyses were performed. TMHMM analysis predicted that 71.6% of these differentially expressed proteins were localized in the outer membrane region and predicted that only 0.9% of the proteins were localized toward the inside of the membrane (Fig. 6b and Additional file 2: Table S3). Furthermore, 27.5% of the proteins exhibited transmembrane helices, and 10 had more than two transmembrane helices. This indicated that these proteins were localized or associated with the bacterial membrane (Fig. 6b and Additional file 2: Table S3). SignalP, SecretomeP, and TatP analyses predicted that 65% of the differentially expressed proteins (71 proteins out of 109) were secretory in nature: (**i**) 62.0% were predicted to secrete through classical pathway, (ii) 35.2% through non-classical pathway, and (iii) 2.8% through bacterial-specific twin-arginine translocation secretion pathway (Fig. 6c and Additional file 2: Table S3). In addition, five proteins (XC_0253, XC_1519, XC_2148, XC_3476, and XC_4152) were discovered to contain signal peptidase II cleavage sites (see discussion hereafter) and, according to DOLOP, a database of bacterial lipoproteins in which 101 lipoproteins in the genome sequence of *X. campestris* pv. *campestris* strain 8004 were identified [45], were predicted to be lipoproteins. Notably, except for XC_2148, the other four putative lipoproteins (XC_0253, XC_1519, XC_3476, and XC_4152) were significantly more abundant in the wild type than the *lolA* mutant (Table 1 and Additional file 2: Table S3). The expression levels of genes encoding these five putative lipoproteins were determined in Xcc17 and H27 by RT-qPCR (see following section).

**Table 1:** Putative lipoproteins, TonB-dependent receptor family, and virulence- and biofilm-related proteins in which expression was differentially altered after *lolA* mutation^a^

Gene ID^b^	Protein name	Cellular component	Biological process	Molecular function	Fold change^c^ (H27/Xcc17)	RT-qPCR^d^ (H27/Xcc17)
Putative lipoprotein with signal peptidase II cleavage site						
XC_0253	Dipeptidyl anminopeptidase			serine-type peptidase activity	†	0.58
XC_1519	Alkaline phosphatase			phosphatase activity	0.40	0.69
XC_2148	Metallopeptidase			metalloendopeptidase activity	‡	1.41
XC_3476	Uncharacterized protein				†	0.39
XC_4152	Cytochrome C biogenesis protein	cell	cell redox homeostasis	antioxidant activity oxidoreductase activity	†	0.30
TonB-dependent receptor family						
XC_0124	TonB-dependent receptor	cell outer membrane	transport	receptor activity	†	0.42
XC_0687	TonB-dependent receptor	cell outer membrane	transport	receptor activity	0.34	0.31
XC_0806♦	TonB-dependent receptor	cell outer membrane	transport	receptor activity	†	0.40
XC_1546	TonB-dependent receptor	cell outer membrane integral component of membrane	transport	receptor activity	0.26	0.35
XC_1644	TonB-dependent receptor	cell outer membrane integral component of membrane	transport	receptor activity	2.30	0.59
XC_2194	TonB-dependent receptor	cell outer membrane	transport	receptor activity	†	0.09
XC_2899	TonB-dependent receptor	cell outer membrane	transport	receptor activity	†	0.51
XC_3063	TonB-dependent receptor	cell outer membrane	transport	receptor activity	0.31	0.38
XC_0558	Ferric pseudobactin M114 receptor protein	cell outer membrane	siderophore transport	iron ion binding receptor activity	0.49	0.33
XC_3559▴	Putative siderophore receptor	cell outer membrane integral component of membrane	siderophore transport	iron ion binding receptor activity	†	0.80
Virulence-related protein						
XC_0626•	Glucanase		cellulose catabolic process	hydrolase activity, hydrolyzing O-glycosyl compounds	†	0.61
Gene ID^b^	Protein name	Cellular component	Biological process	Molecular function	Fold change^c^ (H27/Xcc17)	RT-qPCR (H27/Xcc17)
XC_0639♦	Endoglucanase		cellulose catabolic process	carbohydrate binding cellulase activity	0.42	0.62^#^
XC_1632♦	VirB8 protein	integral component of membrane		oxidoreductase activity	†	0.52
XC_1806•	Virulence regulator	intracellular	regulation of transcription, DNA-templated	DNA binding	0.49	0.83
XC_3540•	Uncharacterized protein				0.43	0.0.57
XC_3696♦	Uncharacterized protein				†	0.27
Biofilm-related protein						
XC_1921	Uncharacterized protein				†	0.78
XC_3686	Uncharacterized protein	integral component of membrane			†	0.87
XC_4290	Hemagglutinin	integral component of membrane	cell communication	calcium ion binding	0.32	0.20^#^

^a^: Detailed information is provided in Additional file 2: Table S3.

^b^: Gene ID is based on *X. campestris* pv. *campestris* strain 8004. ♦, ▴, and • indicate proteins have been reported previously to be virulence-related in *X. campestris* pv. *campestris*, *X. oryzae* pv. *oryzicola*, and *X. oryzae* pv. *oryzae*, respectively.

^c^: † and ‡ indicate the protein is unique to the wild type Xcc17 and the *lolA* mutant H27, respectively.

^d^: ^#^ indicates the previously tested genes presented Fig. 4.

**Fig. 7:**
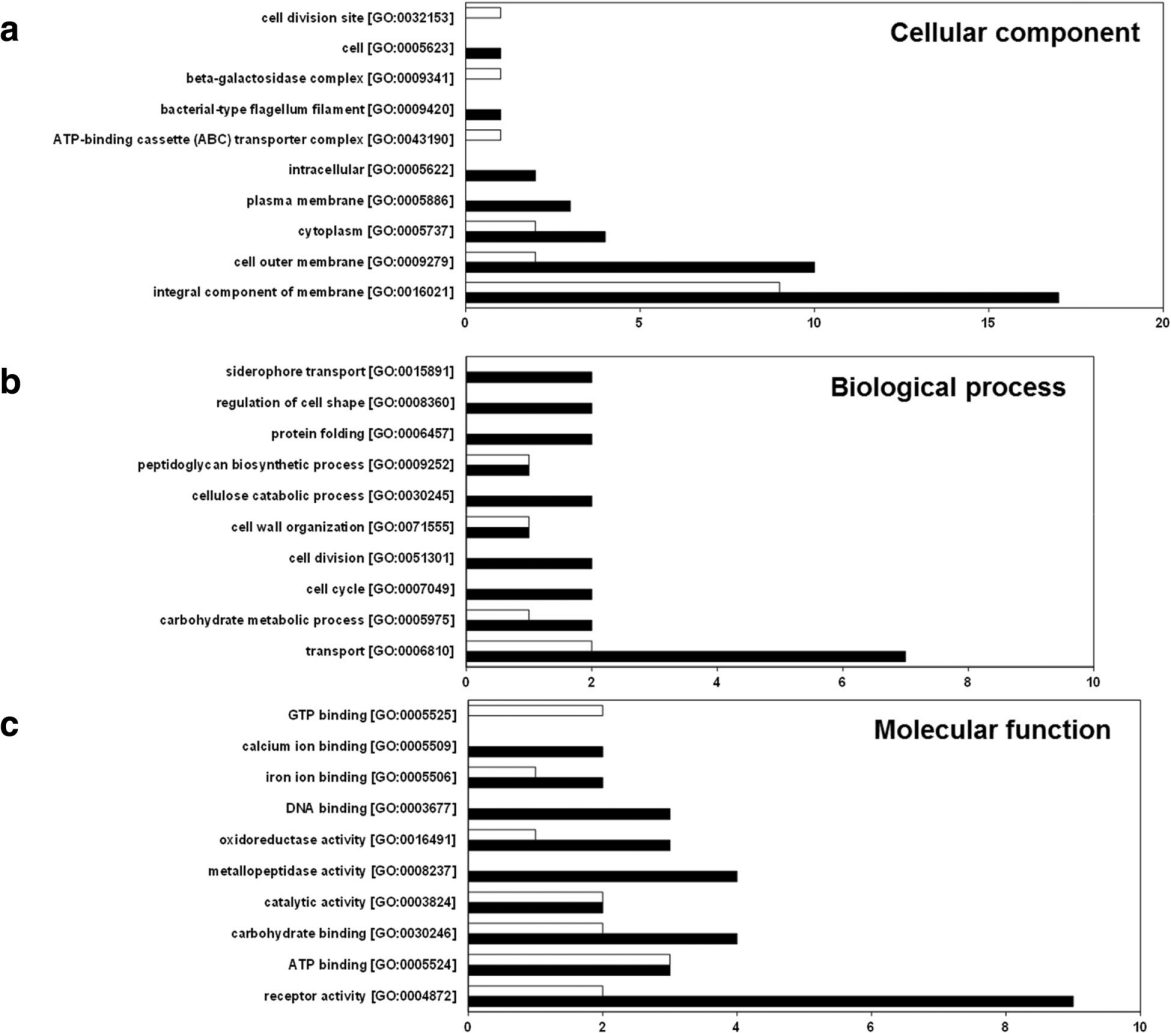
GO term analysis of differentially expressed proteins that were identified using the proteomic approach classified according to cellular component category (**a**), biological process category (**b**), and molecular function category (**c**). The black bar and white bar denote proteins in which the expression levels were upregulated and downregulated in Xcc17, respectively. Numbers of proteins in each category are presented on the X-axis. Detailed information concerning the differentially expressed proteins that were identified can be found in Additional file 2: Table S3

To further investigate these differentially expressed proteins, they were categorized on the basis of their GO term annotation using DAVID functional annotation tools and were categorized according to their annotated function with respect to biological processes, molecular functions, and cellular components,. Results of the top ten GO analyses for the differentially expressed proteins are presented in Fig. 7, and details are displayed in Additional file 2: Table S3. GO analysis revealed that 46, 46, and 70 differentially expressed proteins were annotated to cellular component, biological process, and molecular function, respectively (Additional file 2: Table S3). The GO terms integral component of membrane (GO:0016021) and cell outer membrane (GO:0009279) in the GO annotation category cellular component (Fig. 7a), transport (GO:0006810) in the biological process (Fig. 7b), and receptor activity (GO:0004872) in the molecular function category (Fig. 7c) are the primary categories of significantly enriched differentially expressed proteins. Most of the differentially expressed proteins belonging to these categories were highly expressed in the wild-type Xcc17 compared with the mutant strain H27. These findings suggested that the *lolA* mutation influenced proteins annotated to be membrane associated (an integral component of a membrane or cell outer membrane), to be involved in transport, or to have receptor activity. Additionally, several biological processes, such as the carbohydrate metabolic process (GO:0005975) and the cellulose catabolic process (GO:0030245), were detected (Fig. 7b). Furthermore, proteins with carbohydrate binding [GO:0030246], catalytic activity [GO:0003824], metallopeptidase activity [GO:0008237], and oxidoreductase activity [GO:0016491] were discovered (Fig. 7c). These findings suggested that these biological processes and molecular functions were affected after *lolA* mutation. Overall, various categories were associated with *lolA* mutation, which indicated a substantial overall phenotypic difference resulting from the *lolA* mutant H27 compared with the wild-type strain Xcc17. Clearly, *lolA* had a broad physiological role in *X. campestris* pv. *campestris*.

### Validation of proteomic results through RT-qPCR

Proteomic analyses indicated the possibility that the *lolA* gene was involved in the expression of proteins associated with the following: the membrane (integral component of membrane [GO:0016021] or cell outer membrane [GO:0009279] in the cellular component category), transport (transport [GO:0006810] in a biological process category), and receptor activity (receptor activity [GO:0004872] in terms of molecular function). Most of the differentially expressed proteins assigned to these GO categories belonged to the TonB-dependent receptor family (XC_0124, XC_0558, XC_0687, XC_0806, XC_1546, XC_1644, XC_2194, XC_2899, XC_3063, and XC_3559) (Table 1 and Additional file 2: Table S3). Among them, the orthologues of XC_0806 (XCC3358) and XC_3559 (XOO0785, PrhA) have been reported to play a role in pathogenicity in the *X. campestris* pv. *campestris* strain ATCC33913 [46] and the *X. oryzae* pv. *oryzicola* strain RS105 [47], respectively. Here, the expression levels of genes encoding these aforementioned proteins were determined in Xcc17 and H27 through RT-qPCR.

Among the differentially expressed proteins, it was also found that: (i) two proteins [VirB8 (XC_1632) and hypothetical protein (XC_3696)] were reported to be involved in the pathogenesis of *X. campestris* pv. *campestris* strain 8004 [48], (ii) three proteins [1,4-beta-cellobiosidase (XC_0626), virulence regulator XrvA (XC_1806), and hypothetical protein (XC_3540)] were reported to have homologues (XOO4035, XOO2744, and XOO3855) known to play roles in the pathogenicity of *X. oryzae* pv. *oryzae* strain KACC10331 [49], and (iii) two hypothetical proteins (XC_1921 and XC_3686) were reported to have homologues (XAC2301 and XAC3657) associated with biofilm formation in *X. citri* subsp. *citri* strain 306 [50]. We also selected genes encoding these aforementioned proteins for RT-qPCR validation.

As presented in Table 1, 22 selected genes that encode differentially expressed proteins were verified through RT-qPCR. The various patterns of transcription levels of these tested genes were similar to the results from proteomic analysis except for XC_1644, which exhibited a result contrary to the proteomic analysis.

## Discussion

Transposon mutagenesis has been used to identify pathogenicity-related genes and to conduct functional genomic analysis in several *Xanthomonas* species in which the complete genome sequence is available, such as *X. campestris* pv. *campestris* strain 8004 [48], *X. citri* subsp. *citri* strain 306 [50–52], and *X. oryzae* pv. *oryzae* strain KACC10331 [49]. Previously, we screened an EZ-Tn*5* library containing approximately 1000 Xcc17 mutants for their attachment ability and identified two novel biofilm-related genes (*prc* and *wxcX*) that have been further determined to play a role in pathogenicity [13, 29]. In this study, we extended our previous work to identify an attachment-reduced mutant (H27) that had a transposon inserted in the *lolA* gene and putatively encoded an outer membrane lipoprotein chaperone in Xcc17. In addition to the recently completed genome sequence of Xcc17 [15], the *lolA* gene has been discovered in several sequenced *X. campestris* pv. *campestris* strains, such as strain ATCC33913 [53], strain 8004 [48], and strain B100 [54]. Sequence comparison revealed that the gene product of Xcc17 *lolA* was identical in both size and amino acid sequence to LolAs from these *X. campestris* pv. *campestris* strains. The orthologous gene of *lolA* was also highly conserved in other sequenced *Xanthomonas* species, such as *X*. *axonopodis* pv. *citrumelo* F1 [55], *X. campestris* pv. *vesicatoria* 85–10 [56], *X. citri* subsp. *citri* (formerly *X. axonopodis* pv. *citri*) 306 [53], *X. gardneri* ICMP 7383 [57], *X. oryzae* pv. *oryzae* KACC10331 [58], and *X. oryzae* pv. *oryzicola* BLS256 [59], and exhibited over 90% amino acid identity. Although the *lolA* gene exists in many *Xanthomonas*, nothing is known of its biological functions, and no relevant reports were discovered in the literature. Here, we provided conclusive genetic evidence demonstrating that *lolA* is required for biofilm formation (Fig. 1), extracellular enzyme synthesis (Fig. 3), and virulence (Fig. 2) of *X. campestris* pv. *campestris*. To our knowledge, this is the first report to establish the participation of the *lolA* gene product in multifarious pathogenicity-related functions, such as biofilm formation, virulence factor production, and pathogenesis. The RT-qPCR results (Fig. 4) indicated that mutation in *lolA* reduced the expression of genes known to be involved in virulence and extracellular enzyme production (*engA*, *manA*, *pelA*, and *prt1*) and bacteria attachment (*galE*) and also reduced the expression of adhesion-related genes (*ha*, *xadA*, and *yapH*) whose involvement in virulence and attachment in *X. campestris* pv. *campestris* is still not known. It is implying that *lolA* might affect the expression of these genes at the transcriptional level and that the reduced extracellular enzyme synthesis and bacterial attachment of the *lolA* mutant may be attributable to the reduced expression of these genes. As a putative outer membrane lipoprotein chaperone, the annotated product encoded by *lolA* is not a regulatory protein, and LolA likely affects the transcription of these virulence-related genes indirectly. LolA possibly affects these genes through an unknown regulatory mechanism such as a two-component signal transduction system (see discussion hereafter) in *X. campestris* pv. *campestris*, but this requires further investigation. In addition, it is also possible that *lolA* mutant might reveal growth defect in plant host and the reduced lesion observed in cabbage might be a consequence of impaired growth of the *lolA* mutant. As the effect of *lolA* mutation on the growth of *X. campestris* pv. *campestris* in host plant has not been evaluated, the possibility that deletion of *lolA* might impact the bacteria to proliferate well and to attain full virulence cannot be excluded. It is worthy of further evaluation.

The LolA homologue is present in multifarious bacteria, and there are 2125 sequences with LolA domain are listed in the Pfam family database [60]. Among them, only the LolAs of *E. coli* and *P. aeruginosa* have been characterized. In *E. coli*, LolA depletion caused a severe growth defect and impaired the outer membrane localization of lipoproteins [61], and the accumulation of outer-membrane-specific lipoproteins in the inner membrane resulting from LolA depletion could likely cause a perturbation of the inner membrane [62]. Through mutagenesis analysis, Arg and Phe at positions 43 and 47 (located in a highly conserved sequence, KRPNLF), respectively, of the *E. coli* LolA were determined to affect abilities to accept and transfer lipoproteins [63–65]. A short helix in the C-terminal region of the *E. coli* LolA was also discovered to have a major role in lipoprotein localization [66]. In *P. aeruginosa*, it appeared that (i) LolA was important for cell growth; (ii) LolA-deficient cells were much more sensitive to the lytic effect of SDS and played a relevant role in cell envelope stability; and (iii) LolA-depleted cells were partially impaired in pathogenicity [67]. The crystal structure of the LolA of *P. aeruginosa* exhibited the presence of several hydrophobic patches on the protein surface, and some amino acid residues in these hydrophobic areas (Trp87, Phe193, and Val195 in the largest hydrophobic patch) were determined to be essential for lipoprotein binding [68]. Our growth analysis indicated that the *lolA* mutant nevertheless grew satisfactorily as the wild type in basal media (data not shown), which suggested that *lolA* is not strictly essential for cell growth in *X. campestris* pv. *campestris*. This differs from situations observed in the *lolA* mutant of *E. coli* and *P. aeruginosa* where *lolA* was required for growth in these two bacteria [61, 67]. Although no differences were evident between the wild type and the *lolA* mutant with respect to the ability to grow in the basal medium, the growth of the *lolA* mutant strain was significantly impeded in the presence of agents that affect cell membrane integrity compared with that of the wild-type strain (Fig. 5). The *X. campestris* pv. *campestris* LolA may be involved in cell envelope stability similar to those observed in *E. coli* and *P. aeruginosa*. This study’s results suggested that the reduced biofilm formation attributable to the *lolA* mutation is caused by altered cell membrane integrity that subsequently affects attachment ability. Because the cell envelope is a protective barrier at the frontline of interactions with the environment, the impaired tolerance against several stresses might be because of the altered cell membrane integrity. Sequence analysis indicated that the aforementioned residues required for LolA function in *E. coli* and *P. aeruginosa* were not entirely conserved in Xcc17 LolA. The conserved residues included (**i**) Phe68 (corresponding to Phe47 in *E. coli* LolA) and (ii) Trp88 and Phe196 (corresponding to Trp87 and Phe193 in *P. aeruginosa* LolA). The residues in comparable positions for Arg43 in *E. coli* LolA and Val195 in *P. aeruginosa* LolA were substituted by Thr64 and Pro198 in Xcc17 LolA, respectively. Although the *lolA* mutant exhibited high sensitivity to the membrane-perturbing compounds EDTA, SDS, and polymyxin B, which might be correlated with its predicted role in lipoprotein outer membrane localization, on the basis of sequence analysis, we cannot definitively conclude that *X. campestris* pv. *campestris* LolA is responsible for lipoprotein outer membrane localization. Overall, the results of the phenotypic evaluation and sequence comparison demonstrated that the *X. campestris* pv. *campestris* LolA played relevant roles in bacterial attachment, virulence factor synthesis, and pathogenicity. In addition, LolA was associated with cell envelope stability, although the mechanism through which it acts on lipoprotein outer membrane localization in *X. campestris* pv. *campestris* remains to be experimentally elucidated.

Proteomic analysis is a powerful method for elucidating the function of genes and proteins, especially for an organism whose genome sequence is established. Consequently, proteomics combined with genomic information can directly and efficiently characterize gene function at the protein level [69]. Although several *X. campestris* pv. *campestris* strains have been sequenced, few proteomic studies focused on this bacteria have been published. Such studies have examined the extracellular proteome of strain B100 [70], undertaken qualitative and comparative proteomic analysis of strain Xcc17 [71], conducted outer-membrane-vesicle-associated protein analysis of strain B100 [72], conducted phosphorylated cytoplasmic protein analysis of strain B100 [73], and performed proteomic analysis of strain 8004 and its *purC* mutant [74]. Here, we employed a quantitative proteomic approach, coupling nLC to MS/MS, to compare the outer-membrane-enriched proteins from the *lolA* mutant and the wild type to elucidate the effects of *lolA* on proteins that are associated with the membrane. Through comparative proteomics analysis, we discerned 71 and 38 proteins that had various putative biological functions and that were present in relatively low and high levels in the *lolA* mutant, respectively, compared with the wild-type strain (Additional file 2: Table S3). Subsequent localization prediction and GO term annotation of the identified differentially expressed proteins were performed, several valuable discoveries were made, and several unanswered questions remain.

Localization prediction analysis determined that most of the differentially expressed proteins that were identified were membrane associated (Fig. 6), which indicated the efficacy of the protein extraction procedure. GO analysis revealed that the differentially expressed proteins that were identified were mainly assigned for association with the membrane or were located in the outer membrane in terms of cellular components (Fig. 7a), which was consistent with the localization prediction analysis. DOLOP predicted that five of the identified proteins were potential lipoproteins [45]; among these, four were upregulated by LolA. The effects of *lolA* mutation on their expression were further validated through RT-qPCR, and a transcriptional level effect was confirmed (Table 1). None of these putative lipoproteins had been characterized with respect to lipid modification and membrane localization. In *E. coli*, lipoproteins are localized in either the inner or the outer membrane, depending on the sorting signal [19]. The inner membrane retention signal (Asp at position 2, + 2 rule) may function as a Lol-avoidance signal and inhibit lipoprotein recognition by LolCDE, resulting in their retention in the inner membrane [19]. Analysis of the predicted amino acid sequences indicated that they had a typical *N*-terminal lipoprotein signal peptide, and the predicted signal peptidase II cleavage site was at LSA^16^–C^17^S for XC_0253, at VAA^20^–C^21^A for XC_1519, at LSA^19^–C^20^K for XC_2148, at LSG^24^–C^25^A for XC_3476, and at LAA^15^–C^16^I for XC_4152. None of these contained the Asp at position + 2 after signal peptide cleavage, suggesting that they are located in the outer membrane. Further study is necessary to clarify this location.

GO term analysis revealed that the functional categories of the differentially expressed proteins that were identified were mainly involved in transport or receptor activity (Fig. 7b and c). In gram-negative bacteria, the outer membrane constitutes a major permeability barrier and is the location of myriad integral transmembrane proteins with β-barrel conformation as well as lipoproteins [75, 76]. Outer membrane proteins play key roles in the structural integrity of the outer membrane and function as transporters, membrane pores, membrane-bound enzymes, or components of signal transduction cascades in gram-negative bacteria [11]. An uptake of nutrients from the surrounding environment mediated by the transporter proteins is necessary for bacteria to survive and adapt under myriad environmental changes. Proteins associated with the membrane or related to the transport system are likely employed by *X. campestris* pv. *campestris* to acquire nutrients from environments and adapt to environmental assaults. The altered expression of these proteins in the *lolA* mutant that causes changes in outer membrane components and the resultant membrane protein imbalance might cause insufficient nutrient uptake, impaired stress tolerance, and a diminished ability to cause disease. Notably, we discovered that *lolA* was involved in the expression of proteins belonging to the TonB-dependent receptor family, and nine of ten proteins were downregulated in the *lolA* mutant strain (Table 1 and Additional file 2: Table S3). TonB-dependent receptors are outer membrane proteins mainly known for their role in the active transport of iron siderophore complexes in gram-negative bacteria [46]. Genome sequence analysis predicted 72 TonB-dependent receptors in *X. campestris* pv. *campestris*, and systematic analysis suggested that some were involved in utilization of plant carbohydrates during infection [46]. *XCC3358*, the orthologue of *XC_0806* in *X. campestris* pv. *campestris* strain ATCC33913, was reported to be required for sucrose transport; its inactivation resulted in impaired virulence in cabbage [46]. In *X. oryzae* pv. *oryzicola*, *prhA* (*XC_3559* orthologue) mutation resulted in impaired virulence in adult rice [47]. Because the biological functions of most of the aforementioned proteins that belong to the TonB-dependent receptor family in *X. campestris* pv. *campestris* have been inadequately investigated, these proteins represent favorable candidates for further analysis in future studies.

In addition to proteins belonging to the TonB-dependent receptor family, our proteomic analysis revealed that the quantities of several proteins, which had been reported to be virulence- or biofilm-related in *Xanthomonas*, were reduced after *lolA* mutation (Table 1). The three virulence-related proteins known to contribute to *X. campestris* pv. *campestris* pathogenesis are XC_0639, XC_1632, and XC_3696. [48]. Studies have concluded that XC_0639 (endoglucanase), the major extracellular cellulase of *X. campestris* pv. *campestris*, plays a role in plant tissue maceration and is essential for pathogenesis [5, 7]. Genes encoding XC_1632 (VirB8, channel-forming protein) and XC_3696 (hypothetical protein) were reported to be involved in virulence [48], but no detailed studies on these two genes have been conducted. The orthologous proteins of XC_0626, XC_1806, and XC_3540 were assigned as virulence factors of *X. oryzae* pv. *oryzae* [49], and the orthologues of XC_1921, XC_3686, and XC_4290 were reported to be associated with biofilm formation of *X. citri* subsp. *citri* [50]. Whether these proteins are also implicated in virulence, biofilm formation, or both in *X. campestris* pv. *campestris* remains unclear, and further clarification of the potential roles they play would be valuable. In addition, only proteins extracted from the enriched outer membrane fraction were investigated. Whether *lolA* is involved in the production of cytoplasmic or extracellular proteins remains to be determined.

Lipoproteins are peripherally anchored membrane proteins that play key roles not only in basic bacterial physiology, such as envelope stability, cell division, transport, and protein folding, but also in bacterial pathogenic mechanisms such as adhesion and colonization [77]. Proper localization of these lipoproteins is vital for their function, and the Lol system is responsible for transferring the lipoproteins from the inner membrane to the outer membrane [16–20]. The mechanisms by which the Lol system sorts and localizes lipoproteins in the outer membrane are satisfactorily understood in *E. coli* [19]. LolA may play a critical role in both the sorting and outer membrane localization of lipoproteins. It interacts with LolCDE at the step of lipoprotein release to accept lipoproteins from LolCDE in the inner membrane; moreover, it interacts with LolB to transfer lipoproteins to LolB [64, 65]. Inhibition of the Lol pathway through LolA depletion or overexpression of the dominant-negative LolA mutant activates the Cpx and Rcs systems, both of which are envelope stress response systems of *E. coli* [62, 78]. The Cpx system is a classical two-component signal transduction system comprising CpxA and CpxR, and it responds to periplasmic or inner membrane protein misfolding, regulates genes crucial for dealing with protein misfolding, performs transport functions, and performs other inner-membrane-associated functions [16, 75, 76]. A study has identified the Cpx stress response as a monitor of lipoprotein trafficking, which is tasked with protecting the cell from mislocalized lipoproteins [79]. The Rcs system, containing RcsA through RcsD and RcsF, is induced by mutations or conditions that can disrupt the cell envelope and regulates genes involved in various cell-surface-related processes [16, 76]. Neither homologous proteins of the Cpx and Rcs systems nor an envelope stress-response system have been identified in *X. campestris* pv. *campestris*, but 106 genes have been predicted to encode two-component signal transduction in the genome sequences in this bacteria [80]. An unknown regulatory pathway, or pathways, parallel to the *E. coli* Cpx and Rcs systems likely exist in *X. campestris* pv. *campestris*, and this pathway, or pathways, might be encoded by the two-component signal transduction genes. LolA likely influences the expression of genes encoding multifarious virulence-related functions by modulating an unknown regulatory pathway, such as the two-component signal transduction system. However, further investigation of the candidate genes that may code for the unknown regulatory pathway that is activated after *lolA* mutation is required to verify the possibility. In *E. coli*, the alternative sigma factor σ^E^, encoded by *rpoE*, is essential for growth and promotes the expression of factors that help to preserve and restore cell envelope integrity to maintain homeostasis of the outer membrane [16, 75, 81]. Additionally, σ^E^ contributes to cell envelope stress adaption in *X. campestris* pv. *campestris* [82]. The effect of *lolA* on σ^E^ has not been documented in *E. coli*, but the possibility that deletion of *lolA* in *X. campestris* pv. *campestris* might be implicated in σ^E^ activity cannot be excluded.

## Conclusion

In conclusion, through genetic complementation and phenotypic evaluation, we acquired evidence that *lolA* plays a role in bacterial attachment, extracellular enzyme synthesis, and pathogenesis of *X. campestris* pv. *campestris* and is necessary for stress tolerance in this cruciferous black rot pathogen. To further investigate the function of *lolA*, a quantitative proteomics approach was used to comprehensively compare the protein profiles of the wild type and the *lolA* mutant. Based on the nLC-MS/MS analysis and bioinformatics predictions, our proteomics results revealed that the abundance levels of 109 proteins were affected by *lolA* mutation, and these differentially expressed proteins belonged to different functional categories. Consistent with phenotypic changes, RT-qPCR analysis revealed that the transcription of major extracellular enzyme genes, attachment-related genes, and several genes previously known to be associated with virulence were reduced in the *lolA* mutant compared with the wild type. To the best of our knowledge, this is the first time that *lolA* has been characterized in *Xanthomonas*, this is the first report to demonstrate that *lolA* is involved in biofilm formation, and this is the first study to conduct proteomic analysis to investigate the role of LolA and obtain novel insights into the function of LolA in bacteria.

## Additional files

Additional file 1: **Table S1.** Primers used for RT-qPCR in this study. (PDF 52 kb)

Additional file 2: **Table S2.** List of the identified proteins from Xcc17 (wild type) and H27 (*lolA* mutant). The gene ID is based on *X. campestris* pv. *campestris* strain 8004. The red and blue colors denote proteins identified in Xcc17 and H27, respectively. **Table S3.** Localization and signal peptide prediction as well as GO analysis of differentially expressed proteins that were identified (fold change > 2.0) from Xcc17 (wild type) and H27 (*lolA* mutant). The † and ‡ denote that the protein is unique to Xcc17 or H27, respectively. The gene ID is based on *X. campestris* pv. *campestris* strain 8004. (XLSX 149 kb)

## Abbreviations

GO: gene ontology
LB: Luria-Bertani
Lol: Lipoprotein outer membrane localization
nLC-MS/MS: Nano liquid chromatography-tandem mass spectrometry
qPCR: Quantitative real-time polymerase chain reaction
RT: Reverse transcription
