# Supplementary material for: Functional characterization and proteomic analysis of lolA in Xanthomonas campestris pv. campestris
Source: BMC Microbiol. 2019 Jan 21;19:20. doi: 10.1186/s12866-019-1387-9 (PMC6341742; doi:10.1186/s12866-019-1387-9)
Supplement: Supplementary file 1 — Table S1. Primers used for RT-qPCR in this study. (PDF 52 kb) [file 12866_2019_1387_MOESM1_ESM.pdf]

Table S1. Primers used for RT-qPCR in this study

Primer pair	Sequence (5′-3′)	Gene ID[a]
**Putative lipoprotein genes**		
0253F/0253R	GCAATTACCAGCTGCGCTAC/TTGGTGACATCCTCGAACGG	XC_0253
1519F/1519R	GCCTACGTGTGGAACGAACA/CGGTCATGGTCGAACTGCAT	XC_1519
2148F/2148R	TGCAGGCTTCGATCACTTGT/GCATCGTCAATCGCACGAAA	XC_2148
3476F/3476R	TGCGCTACCACACCATCATC/CTATGCCAGGACGTCACCAC	XC_3476
4152f/4152R	CGCAATGTGCCATTGGTGAT/CTCCGTGGTATCGAACAGGC	XC_4152
**TonB-dependent receptor genes**		
0124F/0124R	GGTGAGGGCAAGCAGATTTC/AGGTATTGAAGTCGAAGCCG	XC_0124 (*iroN*)
0687F/0687R	CCGGTTATGCCTTGGTGGAT/GGAACACGTCTTCGGCTACT	XC_0687 (*fepA*)
0806F/0806R	CGCATGGGCTACTACACCTC/GGCTCTGCACGTAGGAGTTC	XC_0806 (*fyuA*)
1546F/1546R	TTCGTGGAGAGCACCATTCC/CGATGCTGCTGTAGTCCGAA	XC_1546 (*btuB*)
1644F/1644R	CAACCTGGATACCGAGTGGG/AATTGGCCTGCGAGGATTGA	XC_1644 (*btuB*)
2194F/2194R	GACCTGTCGCTGGAATGGTA/CCTCGATGTCCCGATGGAAG	XC_2194 (*cirA*)
2899F/2899R	CATCCCGACCATCCTCATCG/GGTCAGCCCATAGCCCATAC	XC_2899 (*btuB*)
3063F/3063R	GTGGACGACATCCAGCTCAA/GTCAGCGACAGGTTGGAGAT	XC_3063 (*fyuA*)
0558F/0558R	GACCTGGACCATCGCTTCAA/GAGCTGGCGTACTTGAGGTT	XC_0558 (*pbuA*)
3559F/3559R	TACCGCAATTTCCTGTCGCT/TCCACATCCACCTGCAACAA	XC_3559
**Virulence-related, biofilm related, and extracellular enzyme genes**		
0626F/0626R	ATGCCTTGGTGTGGGGTAAG/CTTGCCCTGGTTGTTGAGGA	XC_0626
0639F/0639R	GTGTGAACGTGTTCGGCTTC/TCATGTCCTTCCAGTTGCGT	XC_0639 (*engA*)
1632F/1632R	ACCGTCATAACAGCTGGTGG/CCATCACAAGGTACGGCACT	XC_1632 (*virB8*)
1806F/1806R	GTGAGGCATCCTCTGCACAT/GCTGCTTGGTGTTGAAGTGG	XC_1806 (*xrvA*))
1921F/1921R	GCGCAACCGTCTGTTTGTAG/CGTTTCGGCATAAGCACTGG	XC_1921
2160F/2160R	GGCAAACGGTGACCTGCACT/GGATCGATGGTGCCGCTGTT	XC_2160 (*yapH*)
2458F/2458R	AGTTCTACATGCGCGACAAC/CGTACATGTGCACGCTGAAA	XC_2458 (*manA*)
3379F/3379R	CGGAACTGGTAAGGGCAGAC/TGGTCGACTTCAACGCTCTG	XC_3379 (*prt1*)
3540F/3540R	TTCCTACGTGGAAGGTGGCT/TCCTTGTTGGCACGCTCATA	XC_3540
3576F/3576R	GCACCTATGAAGCCAACGTG/GCTGAGCGACTGGAAGAACT	XC_3579 (*xadA*)
3591F/3591R	AAGCCGAAATTCTCAACGGC/AACTTCCAGTGCAGGATCGG	XC_3591 (*pelA*)
3686F/3686R	TCGCTTCCAGATCCGTCTTG/CCAGGGAGATTTCGTTGCCA	XC_3686
3696F/3696R	TACAACGTCAGCGGAGGAAC/ACGAGAAACGCAGGAACCTT	XC_3696
3767F/3767R	GCTCCCACACCTGCGTTGAT/CGCACGCACGTCCATGCAAT	XC_3767 (*galE*)
4290F/4290R	GGTACCTAGTCAACCCACGC/CTGGCAACGCTGTAGGTGTA	XC_4290 (*ha*)
**Internal control**		
16SF/16SR	GTA AAGCGTGCGTAGGTGGT/CGTGCCTCAGTGTCAGTGT	XC_4386 (*16S rRNA*)

[a]: Gene ID is based on *X. campestris* pv. *campestris* strain 8004. The gene name is listed in parentheses if available.
